# Effect of genetic polymorphism of brain-derived neurotrophic factor and serotonin transporter on smoking phenotypes: A pilot study of Japanese participants

**DOI:** 10.1016/j.heliyon.2019.e01234

**Published:** 2019-02-15

**Authors:** Masanori Ohmoto, Tatsuo Takahashi

**Affiliations:** Faculty of Pharmaceutical Sciences, Hokuriku University, Ho-3 Kanagawa-machi, Kanazawa 920-1181, Japan

**Keywords:** Genetics, Epidemiology, Neuroscience

## Abstract

**Purpose:**

This study investigated whether a gene polymorphism causing a Val66Met substitution (rs6265) in brain-derived neurotrophic factor (BDNF) is associated with smoking initiation, smoking cessation, nicotine dependence and age of smoking initiation, in Japanese participants. Additionally, this study examined whether the S allele of the serotonin transporter gene-linked polymorphic region (5-HTTLPR) is associated with the *BDNF* Val66Met polymorphism on smoking phenotypes.

**Patients and methods:**

The genotypic proportion of the polymorphism responsible for *BDNF* Val66Met was determined in 148 participants including 88 current smokers, 21 former smokers, and 39 never smokers, and Fisher's exact test was used to investigate the relationship between this polymorphism and smoking cessation and initiation as well as the association between the genotypes of current smokers with a heavy smoking index (HSI) and the age of smoking initiation. In addition to the *BDNF* Val66Met polymorphism, the *5-HTTLPR* polymorphism has also been evaluated in a specific subset of participants.

**Results:**

We found statistically significant correlations between the *BDNF* Val66Met polymorphism and the HSI, both in the whole study sample (P = 0.017) and in the male subgroup (P = 0.049). Moreover, the *5-HTTLPR* polymorphism was associated with the age of smoking initiation in current smokers carrying the *BDNF* Met allele, in both the whole study sample (P = 0.041) and the male subgroup (P = 0.041). On the other hand, no association was observed between the *BDNF* Val66Met polymorphism, either alone or in combination with the *5-HTTLPR* polymorphism, and the age of smoking cessation. Finally, no independent effects of the *BDNF* Val66Met genotype on the age of smoking initiation were detected.

**Conclusion:**

This pilot study provides preliminary findings regarding the influence of *BDNF* Val66Met on smoking phenotypes and the interacting effect of *5-HTTLPR* on the association between *BDNF* Val66Met and smoking phenotypes in Japanese participants.

## 1 Introduction

Recent genome-wide association studies have identified associations between common allelic variants and smoking phenotypes [1, 2]. Notably, the brain-derived neurotrophic factor (BDNF) Val66Met (rs6265) polymorphism has been found to be strongly related to smoking initiation, as reported by the Tobacco and Genetics Consortium [3]. High levels of BDNF mRNA are expressed in dopaminergic neurons projecting from the ventral tegmental area to the nucleus accumbens [4]. Regarding function, BDNF specifically potentiates dopamine release in the nucleus accumbens through activation of the dopaminergic nerve terminals [5]. Several studies have investigated the associations of *BDNF* Val66Met with smoking behavior and nicotine dependence. Lang et al [6] reported a significantly higher Met allele proportion among smokers than among never smokers in a Caucasian sample, although the association appeared to be male-sex specific [7]. However, Montag et al [8] were unable to replicate this positive association in large samples of Caucasians.

Similar studies of Asian populations have yielded conflicting results. Following a study of Chinese male volunteers, Zhang et al [9] suggested that *BDNF* Val66Met influences the age at which smoking is initiated but not smoking behaviors or nicotine dependence. In a study of Chinese smokers with schizophrenia, those carrying the Met allele had significantly higher scores of nicotine dependence relative to those with the Val/Val genotype [10]. However, a study of Thai men concluded that *BDNF* Val66Met was unlikely to influence susceptibility to smoking [11]. Accordingly, another Asian population study is needed to examine and confirm the association between *BDNF* Val66Met and smoking phenotypes.

BDNF significantly interacts with serotonin in the context of brain functions [12]. Specifically, BDNF promotes the survival and differentiation of serotonergic neurons, and in turn, serotonergic transmission exerts powerful control over BDNF expression. Serotonergic transmission itself is influenced by the serotonin transporter gene-linked polymorphic region (*5-HTTLPR*), which is characterized by two common variants: a long (L) and a short (S) allele [13, 14]. Lerman et al [15] first hypothesized that the S allele may exert a protective effect against smoking and evaluated the association of smoking behavior with the *5-HTTLPR* genotype but failed to find a significant association. The S allele is associated with high neuroticism, and thus, individuals with an S allele find it more difficult to quit smoking [16]. Gerra et al [17] also demonstrated an effect of S allele on neuroticism in heavy-smoking adolescents. The observed associations between the S allele and neuroticism suggest that this allele may enhance neuroticism and thus mediate nicotine addiction.

To the best of our knowledge, previous reports have not discussed the effect of the gene-gene interaction on smoking phenotypes. In the present study, we, therefore, investigated the hypothesis that *BDNF* Val66Met is associated with smoking cessation and initiation, nicotine dependence and the age of smoking initiation in Japanese participants. Given the lack of clarity surrounding this subject, we also examined the possibility that the *5-HTTLPR* S allele could modify the effects of *BDNF* Val66Met on smoking phenotypes.

## 2 Materials and methods

### 2.1 Participants

Japanese healthy participants were recruited among the students, staff, and their siblings at Hokuriku University. The institutional review committee of Hokuriku University approved this study (H25-10). All participants were informed of the aims and methods of the study and provided both verbal and written consent. Participants were categorized as former smokers if they had quit cigarette smoking at least 1 year prior to the interview. Never smokers were individuals who had never smoked a cigarette in their lifetime. Current cigarette smokers (i.e., current smokers) responded to the survey regarding the number of cigarettes smoked per day, time of the first cigarette of the day and the age at which smoking was initiated. Nicotine dependence was estimated using the Heaviness of Smoking Index (HSI) [18], which was calculated by summing the two scores of the time to smoke the first cigarette of the day after awakening and the number of cigarettes smoked per day. The HSI score is based on a 0–6 scale, and individuals with high nicotine dependence received HSI scores ≥4. In Japan, individuals younger than 20 years cannot purchase cigarettes or tobacco products, and the distributors are legally forbidden to sell tobacco products to them. Therefore, the current smokers were subdivided according to whether they had begun smoking before or after 20 years of age.

### 2.2 Genotyping

The DNA in buccal cells was extracted using a kit (EPICENTRE® Biotechnologies, Madison, WI, USA). *BDNF* Val66Met polymorphisms were amplified with sense (5′-AGGTGAGAAGAGTGATGACC-3′) and antisense (5′-CTGGACGTGTACAAGTCTGC-3′) primers using the MightyAmp DNA Polymerase ver.2 (TaKaRa Bio Inc., Japan); the PCR conditions were as follows: initiation at 98 °C for 2 min, 30 cycles of 10 sec of denaturation at 98 °C, 15 sec of annealing at 60 °C, and 20 sec of extension at 68 °C. The PCR products were then purified by Gen Elute PCR Clean-Up Kit (Sigma-Aldrich Inc., USA) and were then genotyped by direct DNA sequencing with an inner antisense primer (5′- ATCCGAGGACAAGGTGGCTT-3′).

To determine *5HTTLPR* polymorphisms, PCR products were generated using the Tks Gflex DNA Polymerase (TaKaRa Bio Inc., Japan) with sense (5′-ATGCCAGCACCTAACCCCTAATGT-3′) and antisense (5′-GGACCGCAAGGTGGGCGGGA-3′) primers to yield 419 (L allele) or 376 bp (S allele) amplicons, which were resolved on 3.0% agarose gels; the PCR conditions were as follows: initiation at 94 °C for 1 min, 30 cycles of 10 sec of denaturation at 98 °C, 15 sec of annealing at 62 °C, and 15 sec of extension at 68 °C.

The distributions of alleles and genotypes in current smokers, former smokers, never smokers and the whole study sample were tested for Hardy Weinberg Equilibrium (HWE). Genotypes associated with *BDNF* Val66Met or *5HTTLPR* polymorphisms were classified according to Val allele homozygosity and Met allele presence or S allele homozygosity and L allele presence, respectively.

### 2.3 Statistical analyses

We compared the *BDNF* Val66Met genotypic proportions among current, former and never smokers, individuals with high and low nicotine dependence and those who started smoking before or after 20 years of age in order to assess the effect of *BDNF* Val66Met on smoking cessation, nicotine dependence and the age at smoking initiation using the Mann–Whitney U test. In order to assess smoking initiation and cessation, smoking behavior was compared in ever smokers (comprising current and former smokers) versus never smokers, and in current smokers versus former smokers, respectively, according to the procedure described by Munafò et al. [19] Categorical variables were represented by a dominant model based on the *BDFN* Met allele (i.e., the presence or absence of the Met allele), according to the report of Zhang et al. [9] Ishikawa et al [20] suggested that individuals with the S/S genotype are less prone to smoke compared to those with the L allele (L/L and L/S genotypes) in a Japanese population. To verify the existence of an interaction between the *BDNF* Val66Met and the *5HTTLPR* polymorphisms with respect to smoking behavior, we first assessed in individuals harboring the *BDNF* Met/Met genotype; these proportions were also subjected to the chi-squared tests to determine HWE. Fisher's exact test was performed to examine the associations of genotype with smoking status and nicotine dependence. A P value <0.05 and a 95% confidence interval (CI) that did not include a value of 1.0 were considered to indicate statistical significance. Associations were further expressed as odds ratios (OR) with a 95% CI. Statistical analysis was performed using Microsoft Excel (2010) and Easy R (ver. 1.32) [21].

## 3 Results

A total of 148 Japanese participants were enrolled in our study (Table 1), including 88 current smokers (men, 81; women, 7), 21 former smokers (men, 19; women, 2), and 39 never smokers (men, 27; women, 12). In the whole study sample, the average ages of current and former smokers were 31.73 years (range: 20–66 years, standard deviation: 12.19 years) and 49.24 years (range: 23–64 years, standard deviation: 10.11 years), respectively. In the male subgroup, the average ages of current and former smokers were 31.53 years (range: 20–66 years, standard deviation: 12.36 years) and 49.26 years (range: 23–64 years, standard deviation: 10.26 years), respectively. Both in the whole study sample and in the male subgroup, current and former smokers significantly differed with respect to age (*P* < 0.01, Mann–Whitney U test). No information was available as to the age of never smokers. Because female participants accounted for 14.2% (n = 21) of the study sample (n = 148) and only 8.3 % (n = 9) of the ever smokers (n = 109), the analyses were conducted, in parallel, in the whole study sample and in the male subgroup to address the skewed sex ratio of the study population. Regarding the HSI, a measure of the degree of nicotine dependence (range: 0–6), the mean scores and standard deviations for current smokers in the whole study sample and the male subgroup were 2.22 ± 1.67 and 2.26 ± 1.66, respectively. Regarding the age at smoking initiation, the means and standard deviations of current smokers in the whole study sample and male subgroup were 19.23 ± 1.89 years (range: 12–27) and 19.16 ± 1.91 years (range: 12–27), respectively.

**Table 1:** Participants' profiles regarding age at the time of the study, age at smoking initiation and heavy smoking index scores.

	Current smokers (n = 88)		Former smokers (n = 21)		Never smokers (n = 39)	
	All	Male	All	Male	All	Male
Number	88	81	21	19	39	27
Age (years)^a^	31.73 ± 12.19^b^	31.53 ± 12.36^c^	49.24 ± 10.11	49.26 ± 10.26	－	－
Age at smoking initiation (years)^a^	19.23 ± 1.89	19.16 ± 1.91	－	－	－	－
HSI^d^	2.22 ± 1.67	2.26 ± 1.66	－	－	－	－

a Mean age (years) ± standard deviation in the whole study sample (All), and in the male subgroup (Male).

b Age difference (P < 0.01) between current and former smokers in the whole study sample (Mann-Whitney U test).

c Age difference (P < 0.01) between current and former smokers in the male subgroup (Mann-Whitney U test).

d Heavy Smoking Index score. The values after the ± symbols indicate a standard deviation.

Table 2 shows the genotype proportions of the *BDNF* Val66Met polymorphism among the current, former and never smokers. The genotype proportions of the *5-HTTLPR* polymorphism among the 66 current, 14 former and 27 never smokers carrying the *BDNF* Met polymorphism are shown in Table 3. The genotypic distributions of the *BDNF* and *5HTTLPR* genes did not significantly deviate from HWE among current smokers, former smokers, never smokers, and the whole study sample. In addition, the allelic proportion of *BDNF* Val66Met in the whole study sample was similar to the proportions observed in previous Japanese population studies [22, 23]. A previous study reported respective *5-HTTLPR* S and L allele proportions of approximately 80% and 20% in a Japanese population [24], similar to the results of our study.

**Table 2:** Allele and genotype proportions of BDNF Val66Met in the whole study sample. Data are presented as numbers and proportions of participants.

Allele or genotype	All (n = 148)^a^		Current smokers (n = 88)		Former smokers (n = 21)		Never smokers (n = 39)	
Val/Val	41	27.7%	22	25.0%	7	33.4%	12	30.8%
Val/Met	82	55.4%	48	54.5%	10	47.6%	24	61.5%
Met/Met	25	16.9%	18	20.5%	4	19.0%	3	7.7%
P value^b^	0.140		0.382		0.899		0.061	
Val	164	55.4%	92	52.3%	24	57.1%	48	61.5%
Met	132	44.6%	84	47.7%	18	42.9%	30	38.5%

a Total number of genotypes.

b Compliance of allelic distribution with the Hardy–Weinberg equilibrium for current smokers, former smokers, never smokers and the whole study sample.

**Table 3:** Allele and genotype proportions of 5-HTTLPR polymorphism in BDNF Met allele carriers. Data are presented as numbers and proportions of participants.

Allele or genotype	All (n = 107)^a^		Current smokers (n = 66)		Former smokers (n = 14)		Never smokers (n = 27)	
S/S	60	56.1%	41	62.1%	8	57.1%	11	40.7%
S/L	40	37.4%	21	31.8%	5	35.7%	14	51.9%
L/L	7	6.5%	4	6.1%	1	7.1%	2	7.4%
P value^b^	0.924		0.559		0.859		0.386	
S	160	74.8%	103	78.0%	21	75.0%	36	66.7%
L	54	25.2%	29	22.0%	7	25.0%	28	33.3%

a Total number of genotypes.

b Compliance of allelic distribution with the Hardy–Weinberg equilibrium for current smokers, former smokers, never smokers and the whole study sample.

No association was observed between the *BDNF* Val66Met proportion and smoking cessation (Table 4) or smoking initiation (Table 5) in either the whole study sample or the male subgroup. Among *BDNF* Met carriers, the proportion of former smokers harboring the S/S polymorphism in *5-HTTLPR* did not differ from those of current smokers in both analyses (Table 6). Moreover, no statistically significant differences were observed in the proportion of *5-HTTLPR* allelic variants between ever smokers and never smokers (Table 7). Therefore, *BDNF* Val66Met may not affect smoking cessation and initiation, and *5-HTTLPR* polymorphism was not found to influence those associations.

**Table 4:** Odds ratios for BDNF Val66Met genotypes in current and former smokers. Each analysis was performed for the whole study sample (left side) and the male subgroup (right side).

Genotype	Smoking cessation^a^	OR (95% CI)	P value^b^
Val/Met, Met/Met	14/66, 12/63	0.67 (0.24–1.86), 0.49 (0.17–1.43)	0.425, 0.239
Val/Val	7/22, 7/18		

a Smoking cessation: number of former smokers per current smokers. OR, odds ratio; CI, confidence interval.

b P values were calculated using Fisher's exact test.

**Table 5:** Odds ratios for BDNF Val66Met genotypes in never and ever smokers. Each analysis was performed for the whole study sample (left side) and the male subgroup (right side).

Genotype	Smoking initiation^a^	OR (95% CI)	P value^b^
Val/Met, Met/Met	27/80, 20/75	1.23 (0.55–2.73), 0.75 (0.25–2.21)	0.678, 0.794
Val/Val	12/29, 5/25		

a Smoking initiation: number of never smokers per ever smokers (combining current and former smokers). OR, odds ratio; CI, confidence interval.

b P values were calculated using Fisher's exact test.

**Table 6:** Odds ratios for 5-HTTLPR genotypes in current and former smokers with the BDNF Met allele. Each analysis was performed for the whole study sample (left side) and the male subgroup (right side).

Genotype	Smoking cessation^a^	OR (95% CI)	P value^b^
S/S	8/41, 6/38	0.81 (0.25–2.62), 0.66 (0.19–2.27)	0.769, 0.537
S/L, L/L	6/25, 6/25		

a Smoking cessation: number of former smokers per current smokers. OR, odds ratio; CI, confidence interval.

b P values were calculated using Fisher's exact test.

**Table 7:** Odds ratios for 5-HTTLPR genotypes in never and ever smokers with the BDNF Met allele. Each analysis was performed for the whole study sample (left side) and the male subgroup (right side).

Genotype	Smoking initiation^a^	OR (95% CI)	P value^b^
S/S	11/66, 11/61	2.23 (0.95–5.27), 1.28 (0.49–3.37)	0.083, 0.626
S/L, L/L	16/43, 9/39		

a Smoking initiation: number of never smokers per ever smokers (combining current smokers and former smokers). OR, odds ratio; CI, confidence interval.

b P values were calculated using Fisher's exact test.

Next, the HSI scores of current smokers with the Val66Met genotype were compared in both in the whole study sample and the male subgroup (Table 8). Within the group of total smokers (n = 88), the proportion of participants with a low HSI score (<4) was significantly higher among the *BDNF* Met carriers (56 low and 10 high HSI score) than among carriers of the Val/Val genotype (13 low and 9 high HSI score) (OR, 3.88; 95% CI, 1.31–11.46; P value, 0.017). This difference was confirmed, albeit marginally significant, in the male subgroup of smokers (n = 81). In the latter subgroup, the numbers of *BDNF* Met carriers with low and high HSI score were 53 and 10, respectively, whereas the numbers of Val/Val carriers with low and high HSI score were 13 and 9, respectively. (OR, 3.37; 95% CI, 1.05–10.80; P value, 0.049). Among smokers with the Val/Val genotype, the proportion of individuals with a high HSI score was higher than among Met allele carriers (40.9% and 38.9% in the whole study sample and the male subgroup, respectively). However, among current smokers carrying the Met allele (66 and 63 in the whole study sample and the male subgroup, respectively), no association was found between the *5-HTTLPR* polymorphism and the HSI score (proportion of the *5-HTTLPR* S/S carriers in the whole study sample and male subgroup: 34/7 and 31/7, respectively; proportion of S/L or L/L carriers: 22/3 and 22/3, respectively) (Table 9). Consequently, these results indicate that *BDNF* Val66Met has an effect on nicotine dependence, but this effect is not interactive with *5-HTTLPR* polymorphism.

**Table 8:** Odds ratios for BDNF Val66Met genotypes in current smokers according to HSI score. Each analysis was performed for the whole study sample (left side) and the male subgroup (right side).

Genotype	HSI^a^	OR (95% CI)	P value^b^
Val/Met, Met/Met	56/10, 53/10	3.88 (1.31–11.46), 3.37 (1.05–10.80)	0.017, 0.049
Val/Val	13/9, 11/7		

a Heavy Smoking Index (HSI) score: respective numbers of current smokers with low (<4)/high (≥4) HSI scores. OR, odds ratio; CI, confidence interval.

b P values were calculated using Fisher's exact test.

**Table 9:** Odds ratios for 5-HTTLPR genotypes in current smokers according to HSI score with the BDNF Met allele. Each analysis was performed for the whole study sample (left side) and the male subgroup (right side).

Genotype	HSI^a^	OR (95% CI)	P value^b^
S/S	34/7, 31/7	0.66 (0.15–2.84), 0.60 (0.14–2.60)	0.730, 0.727
S/L, L/L	22/3, 22/3		

a Heavy Smoking Index (HSI) score: respective numbers of current smokers with low (<4)/high (≥4) HSI scores. OR, odds ratio; CI, confidence interval.

b P values were calculated using Fisher's exact test.

No significant direct association was found between *BDNF* Val66Met and the age of smoking initiation, either in the whole study sample (n = 88) or in the male subgroup (n = 81) (Table 10). However, among the participants who began smoking before age 20, the proportion of *BDNF* Met carriers was higher (30/36 and 28/35 for the whole study sample and the male subgroup, respectively) than that of Val/Val carriers (5/17 and 5/13, for the whole study sample and the male subgroup, respectively) in both the whole study sample and the male subgroup (ORs 2.83 and 2.08 for total smokers and male smokers, respectively). Moreover, as shown in Table 11, a significant correlation was observed between the *5-HTTLPR* polymorphism and the age of smoking initiation in current smokers carrying the Met allele. Specifically, in the latter subgroup, a significantly higher proportion of early smokers was found among participants homozygous for the *5-HTTLPR* S allele (23/18 and 21/17, for the whole study sample and the male subgroup, respectively) than among carriers of the L allele (7/18 for both the whole study sample and the male subgroup). Odds ratios for the association, in the BDNF Met carriers, between the S/S genotype and early smoking initiation (before age 20), were as follows: whole study sample, OR = 3.29; 95% CI, 1.13–9.57; P value, 0.041; male subgroup, OR = 3.18; 95% CI, 1.08–9.37; P value, 0.041. Therefore, a combined action of the BDNF Val/Met and the *5-HTTLPR* polymorphism may contribute to individual differences in the age of smoking initiation.

**Table 10:** Odds ratios for BDNF Val66Met genotypes in current smokers according to age at smoking initiation. Each analysis was performed for the whole study sample (left side) and the male subgroup (right side).

Genotype	Age at smoking initiation^a^	OR (95% CI)	P value^b^
Val/Met, Met/Met	30/36, 28/35	2.83 (0.94–8.59), 2.08 (0.66–6.54)	0.079, 0.279
Val/Val	5/17, 5/13		

a Age at smoking initiation: numbers of smokers who began smoking at <20 years or ≥20 years of age. OR, odds ratio; CI, confidence interval.

b P values were calculated using Fisher's exact test.

**Table 11:** Odds ratios for 5-HTTLPR genotypes in current smokers according to age at smoking initiation with the BDNF Met allele. Each analysis was performed for the whole study sample (left side) and the male subgroup (right side).

Genotype	Age at smoking initiation^a^	OR (95% CI)	P value^b^
S/S	23/18, 21/17	3.29 (1.13–9.57), 3.18 (1.08–9.37)	0.041, 0.041
S/L, L/L	7/18, 7/18		

a Age at smoking initiation: numbers of smokers who began smoking at <20 years or ≥20 years of age. OR, odds ratio; CI, confidence interval.

b P values were calculated using Fisher's exact test.

## 4 Discussion

Our study demonstrated an association between *BDNF* Val66Met and the HSI, a measure of the degree of nicotine dependence, in our participants. The proportion of smokers who carried the Met allele was significantly higher than that of smokers carrying the Val/Val genotype; the former group had lower HSI scores. However, no significant association between *5-HTTLPR* polymorphism and HSI was observed among Met allele carriers. Accordingly, the *BDNF* Met allele may be associated with reduced nicotine dependence independently of *5-HTTLPR* polymorphism. A previous study of European–American men [7] also demonstrated a significant association between *BDNF* Val66Met and HSI scores. These results could be explained by the negative effect of this polymorphism on mature BDNF secretion. Egan et al. [25] reported that *BDNF* Val66Met affects the intracellular trafficking and packaging of pro-BDNF, thus decreasing the secretion of mature BDNF. The binding of mature BDNF to the TrkB receptor activates multiple intercellular cascades to regulate neuronal development, plasticity and long-term potentiation [26]. Nicotine reinforces smoking addiction by activating the dopaminergic nervous system that projects from the ventral tegmental area to the nucleus accumbens via nicotinic acetylcholine receptors, a process that is affected by the induction of long-term potentiation in these areas [27, 28]. In the current study, we speculated that Met allele carriers exhibit reduced mature BDNF secretion and decreased long-term potentiation relative to smokers with the Val/Val genotype. Consequently, smokers who carry the Met allele may have low nicotine dependence as a result of the effect of nicotine on the dopaminergic system activation.

We did not detect any statistically significant association between *BDNF* Val66Met and smoking cessation and initiation. Furthermore, smoking cessation among *BDNF* Met carriers did not appear to be related to the *5-HTTLPR* polymorphism. Notably, a significant association was found between the *5-HTTLPR* polymorphism and the age of smoking initiation in carriers of the *BDNF* Met allele. Early-age smoking initiation (before age 20) was more frequent in smokers carrying the *5-HTTLPR* S/S genotype than in those with the S/L or L/L genotype. Previously, Zhang et al [9, 10] noted that smokers who carried the Met allele began smoking significantly earlier than those carrying the Val/Val genotype, suggesting that *BDNF* Val66Met may influence the age at which smoking is initiated. A strong association of *BDNF* Val66Met with smoking initiation, but not cessation, was reported by Furberg et al. [3] In contrast, Breetvelt et al [29] suggested that *BDNF* Val66Met is associated with smoking cessation, but not smoking initiation. These authors demonstrated that genetic variation in *BDNF* could alter the reward mechanism by modulating the dopamine reward circuits after an initial nicotine exposure and could thus contribute to altered drug-related memories. In addition, functional magnetic resonance imaging of human participants demonstrated that the Met allele was associated with poorer episodic memory and abnormal hippocampal activation [25]. Regarding *5-HTTLPR*, Nilsson et al [30] reported that in adolescents, the likelihood of a positive smoking status and a higher rate of nicotine dependence was based on the relationship between the *5-HTTLPR* genotype and family environment. For adolescents, a variety of psychosocial factors contribute to smoking [31]. Therefore, genetically vulnerable individuals (i.e., S allele carriers), particularly adolescents, may begin to smoke tobacco impulsively in response to triggers, such as negative moods and environmental factors. Notably, acute immobilization stress has been associated with marked reductions in hippocampal BDNF and raphe nuclei 5HTT mRNA in rats [32]. Hiio et al [33] found that adolescent *BDNF* Met allele carriers along with the *5-HTTLPR* S/S genotype had the lowest conscientiousness scores, suggesting a significant interacting effect of the *5-HTTLPR* and *BDNF* Val66Met polymorphisms on conscientiousness. Therefore, we speculate that the addictive behaviors and personality traits associated with a high risk of smoking initiation may be consistent with a synergistic effect of the *BDNF* Met variant, which reduces BDNF secretion among individuals harboring the S/S genotype of *5-HTTLPR* and is presumed to be impaired with regard to brain 5-HT transmission. Therefore, our finding suggests that the Met allele of *BDNF* Val66Met may promote the initiation of smoking behavior at an early age through interactions with the S/S genotype of *5-HTTLPR*.

This study has several limitations. One major concern pertains to our small sample size. Although Fisher's exact test was adapted to detect any type of significant association in this study, according to a statistical calculator [34], the required sample size would be around 2,000 participants if Pearson's chi-square test was performed with a statistical power of 80% and a small effect size (0.1), which would be expected for the gene's effect on smoking. Additionally, participants were subdivided according to several variables, such as smoking status and gender. None of our significant results survived the Bonferroni correction for multiple hypothesis testing. Furthermore, interpretations of positive results should account for the fact that the P values were not corrected for multiple testing. Therefore, the statistical power does not appear to be adequate, thus raising the possibility of false positivity. Another concern is that the small sample size may have caused selection and confounding biases. A significant difference was observed between the ages of current and former smokers in our analysis of the association between *BDNF* polymorphism and smoking behavior. Additionally, given the very low proportion of female smokers, the analyses were conducted, in parallel, in the whole study sample and in the male subgroup, to overcome the possible problem represented by the skewed sex ratio. No major differences were observed between the whole study sample and the male subgroup. However, the association between *BDNF* Val66Met and changes in the brain has been suggested to be sex-specific [35, 36, 37]. We must also note that personality traits comprise a crucial trigger by motivating people to initiate and continue smoking. Munafò et al [38] demonstrated that participants harboring the S/S and L/L genotypes of *5-HTTLPR* differed significantly with respect to anxiety-related traits. We did not standardize the participants' personality traits, chronic illnesses, particularly neuropsychiatric diseases, or medications, which may have led to bias. Nicotine dependence was assessed on the basis of the HSI, whereas no other measures were employed, such as the Fagerstrom test, pack-year smoking, and smoking years. The smoking status of participants was exclusively based on self-reporting. Furthermore, only two polymorphic variants (*BDNF* Val66Met and *5-HTTLPR*) were assessed. Therefore, our results are of low generalizability, and our findings should be validated in studies with larger samples.

## 5 Conclusion

Our findings indicated reduced nicotine dependence among current smokers who carried the *BDNF* Met allele relative to those homozygous for the Val allele. Moreover, among the Met allele carriers, current smokers homozygous for the *5-HTTLPR* S allele displayed significantly higher rates of smoking initiation before age 20, as compared to those harboring the *5-HTTLPR* L allele. *BDNF* Val66Met had no direct effect on smoking cessation or initiation, and no interactive effect of the *BDNF* Val66Met and the *5-HTTLPR* polymorphisms on smoking cessation was detected. The present study thereby provides preliminary data suggesting potential associations of *BDNF* polymorphism with nicotine dependence and the age at smoking initiation due to an interacting *5-HTTLPR* polymorphism in a small number of Japanese participants.

## Declarations

### Author contribution statement

Masanori Ohmoto: Conceived and designed the experiments; Performed the experiments; Analyzed and interpreted the data; Wrote the paper.

Tatsuo Takahashi: Conceived and designed the experiments; Wrote the paper.

### Funding statement

This work was supported by a general grant to the Faculty of Pharmaceutical Sciences, Hokuriku University. The funding source was not involved in the collection, analysis, interpretation of the data, preparation of the manuscript or the decision to submit the manuscript for publication.

### Competing interest statement

The authors declare no conflict of interest.

### Additional information

No additional information is available for this paper.
